# Near‐Peer Anatomy‐Anchored Teaching

**DOI:** 10.1111/tct.70539

**Published:** 2026-09-30

**Authors:** O. Rackham, M. M. Maitland, P. Ahluwalia, O. Murray, J. E. McKay

**Affiliations:** ^1^ Medical School The University of Edinburgh Edinburgh UK; ^2^ Anatomy Department The University of Edinburgh Edinburgh UK; ^3^ The Usher Institute The University of Edinburgh Edinburgh UK

**Keywords:** anatomy, near‐peer, orthopaedic surgery, preparedness, undergraduate

## Abstract

**Background:**

The transition from pre‐clinical to clinical medicine is challenging, particularly in applying anatomical knowledge to patient care. Reductions in dedicated anatomy teaching time have compounded this. Near‐peer teaching may help address this gap by reducing hierarchy and enhancing psychological safety, though few programmes have explicitly targeted the pre‐clinical to clinical transition through the integration of anatomy with clinical cases.

**Approach:**

A prospective educational evaluation examined a near‐peer, case‐based orthopaedic anatomy programme delivered to Year 4 medical students by Year 5 facilitators across four sessions. Knowledge acquisition was assessed using parallel pre‐ and post‐session multiple‐choice question (MCQ) sets compared using Wilcoxon signed‐rank test and Cohen's *d*_*z*_. Student experience was evaluated using purpose‐designed questionnaires.

**Evaluation:**

Thirty‐two matched pairs were available for analysis. Baseline anatomical confidence (mean 2.76/5) and ratings of placement‐based anatomy teaching (mean 2.26/5) indicated clear unmet need. MCQ scores improved significantly from 5.0 to 7.0 out of 8 (mean 5.00 vs. 6.69, *p* < 0.001, Cohen's *d*_*z*_ = 1.12). Post‐session confidence improved to 4.03/5, facilitators were rated highly (mean 4.59/5), and 97% of students felt comfortable engaging with peer facilitators.

**Implications:**

This programme provides preliminary support for near‐peer, case‐based anatomy teaching as a promising approach to supporting the pre‐clinical to clinical transition, demonstrating short‐term improvements in MCQ performance, confidence and student satisfaction.

## 1 Background

The transition from pre‐clinical to clinical training presents a recognised challenge, particularly in applying foundational anatomical knowledge to patient care [1]. In surgical specialties such as Trauma & Orthopaedics (T&O), failing to address this gap has direct implications, as safe assessment and management rely on anatomical understanding [2]. Dedicated anatomy teaching time fell by 31% across UK and Irish medical schools between 1999 and 2019 [3], leaving students with reduced opportunities to revisit anatomy in clinically relevant contexts. Learning on placement is largely informal and opportunistic, depending on clinician availability, interest and institutional support [4, 5]. Hierarchical clinical learning environments may further limit engagement and disclosure of knowledge gaps [6].

Near‐peer teaching (NPT) may help address these challenges. By reducing hierarchy and increasing cognitive and social congruence, NPT can foster psychological safety and active participation [7, 8]. Although near‐peer anatomy programmes exist [9], few have explicitly targeted the pre‐clinical to clinical transition through integration of anatomy with specialty‐specific cases and imaging interpretation. We therefore designed and evaluated a near‐peer, case‐based orthopaedic anatomy programme aimed at supporting the pre‐clinical to clinical transition while improving anatomical knowledge and confidence.

*By reducing hierarchy and increasing cognitive and social congruence, NPT can foster psychological safety and active participation*.

## 2 Approach

### 2.1 Facilitator Recruitment and Training

Nine Year 5 facilitators were recruited and divided into four groups, each developing a clinical case aligned to UK Medical Licensing Assessment (UKMLA) requirements. Two faculty supervisors, an orthopaedic surgeon (J.E.M.) and an anatomy lecturer (O.M.), provided oversight and reviewed all materials. All facilitators attended a training session on evidence‐informed teaching and received feedback on their teaching from peers and faculty prior to delivery.

### 2.2 Programme Delivery

Four sessions were delivered to Year 4 students (12 per session; total capacity 48). Each 2.5‐h session began with an introduction to orthopaedic imaging, focusing on X‐ray interpretation of relevant joints, followed by case discussions in groups of three to four students. Cases covered rotator cuff tears, hip fractures, supracondylar humerus fractures and distal radius fractures.

### 2.3 Evaluation

Pre‐session questionnaires assessed baseline anatomical confidence and prior NPT exposure (Table 1). Post‐session questionnaires assessed satisfaction, perceived programme benefit and suggestions for improvement (Table 2). Free‐text responses were reviewed by the lead author and used to illustrate student experience; no formal thematic analysis was undertaken.

**TABLE 1:** Pre‐session questionnaire items.

No.	Question	Response format	Scale/options
1	How confident do you feel in your understanding of the clinically relevant anatomy for the topics being taught today?	5‐point Likert	1 = *not at all confident*, 5 = *very confident*
2	How often do you consciously use anatomical knowledge when assessing patients on placement?	5‐point Likert	1 = *never*, 5 = *always*
3	How effective do you feel anatomy teaching is during clinical placements overall?	5‐point Likert	1 = *not at all effective*, 5 = *very effective*
4	To what extent do you feel anatomy is prioritised by clinical supervisors during placement teaching?	5‐point Likert	1 = *not at all*, 5 = *very much*
5	What barriers, if any, prevent you from revisiting anatomy while on placement?	Multi‐select	Lack of time/lack of teaching/low perceived relevance/limited resources/do not know what to revise
6	How useful do you expect an anatomy‐anchored teaching session to be for clinical learning?	5‐point Likert	1 = *not at all useful*, 5 = *extremely useful*
7	Have you previously taken part in sessions led by near‐peer teachers?	Binary (yes/no)	Yes/no
8	What benefits do you anticipate from a near‐peer‐led anatomy session?	Free‐text	Open response

*Note:* All items were designed de novo. Likert scale items: 1 = *lowest*, 5 = *highest*.

**TABLE 2:** Post‐session questionnaire items.

No.	Question	Response format	Scale/options
1	How confident do you now feel in your understanding of the relevant orthopaedic anatomy?	5‐point Likert	1 = *not at all confident*, 5 = *very confident*
2	How much has your understanding improved as a result of this session?	5‐point Likert	1 = *not at all*, 5 = *greatly*
3	How useful was anchoring the session around anatomical principles for understanding clinical presentations?	5‐point Likert	1 = *not at all useful*, 5 = *extremely useful*
4	Did the session help you integrate anatomy into clinical reasoning?	Categorical	Yes, greatly/yes, somewhat/no
5	What features made the anatomy clinically relevant?	Free‐text	Open response
6	How effective were the near‐peer teachers in delivering the content?	5‐point Likert	1 = *not at all effective*, 5 = *very effective*
7	Did near‐peer teaching make you feel more comfortable asking questions or engaging?	Categorical	Yes/somewhat/no
8	What advantages or limitations did you perceive in the near‐peer model?	Free‐text	Open response
9	Do you feel more motivated to revisit anatomy during clinical placement?	Categorical	Yes/somewhat/no/not sure
10	How likely are you to apply the anatomical concepts discussed when seeing patients?	5‐point Likert	1 = *very unlikely*, 5 = *very likely*
11	Overall, how satisfied were you with the session?	5‐point Likert	1 = *very dissatisfied*, 5 = *very satisfied*
12	What aspects of the session were most helpful?	Free‐text	Open response
13	What would you improve for future sessions?	Free‐text	Open response

*Note:* All items were designed de novo. Likert scale items: 1 = *lowest*, 5 = *highest*.

Knowledge acquisition was assessed using parallel sets of eight single‐best‐answer multiple‐choice questions (MCQs) administered immediately before and after each session. Questions were mapped to learning objectives and reviewed by faculty supervisors for content validity. Scores were compared using the Wilcoxon signed‐rank test, with effect size calculated using Cohen's *d*_*z*_. Students were assigned numerical identifiers to enable pairing. Duplicate identifiers and unmatched responses were excluded.

Following completion, facilitators participated in a group debrief. Reflections were summarised and representative comments used to illustrate facilitator experience; no formal thematic analysis was undertaken.

## 3 Evaluation

Following formal ethical approval from the Medical Education Research Ethics Committee, University of Edinburgh (reference: 202629), implied consent was considered sufficient given the nature of data collection.

### 3.1 Baseline Characteristics

Sessions were fully subscribed; 38 of the 48 students attended. Baseline anatomical confidence (mean 2.76/5) and perceived placement‐based anatomy teaching effectiveness (mean 2.26/5) indicated unmet need. Students reported infrequent application of anatomy in clinical settings (mean 2.58/5). Main barriers were lack of teaching (76%) and lack of time (74%). Anticipated usefulness was high (mean 4.00/5), with students expecting teaching accessible to their level, with one anticipating that ‘they know what is relevant to our study and can provide teaching appropriate to our level’, and another expecting ‘a more relaxed atmosphere’.

*They know what is relevant to our study and can provide teaching appropriate to our level*.

### 3.2 Student Experience

Post‐session confidence increased to 4.03/5, perceived improvement in understanding was high (mean 4.44/5), and anatomical anchoring was rated as highly useful (mean 4.32/5). All 34 post‐session respondents reported improved perceived integration of anatomy into clinical reasoning, with one respondent commenting ‘learning the bones, then muscles and nerves and then relating it to clinical cases was really useful’. Facilitators were highly rated (mean 4.59/5), and 97% felt comfortable engaging, with one respondent commenting that ‘it felt like a safer space, [I was] more comfortable learning with other students’. Overall satisfaction was high (mean 4.59/5), with increased motivation to revisit anatomy (71%). Suggested improvements included longer sessions, broader topic coverage and more MCQs.

*Learning the bones, then muscles and nerves and then relating it to clinical cases was really useful*.

### 3.3 Facilitator Experience

Facilitator reflections highlighted the benefit of preparing and delivering sessions for their own learning and exam confidence, with one noting that ‘teaching is so useful for our own learning and knowing what is and isn't relevant [for our own exams]’. Small groups were perceived as particularly conducive to direct engagement and personalised teaching, with one facilitator commenting that ‘small groups meant you could answer questions directly [and] each group could have personalised teaching’.

### 3.4 Knowledge Acquisition

Of 38 students completing pre‐session MCQs, 32 matched pairs were available for analysis; six unmatched responses were excluded due to identifier errors rather than systematic attrition. Median MCQ scores improved from 5.0 to 7.0 out of 8 (mean 5.00 [SD 1.48] vs. 6.69 [SD 1.03], *p* < 0.001, Cohen's *d*_*z*_ = 1.12) (Table 3).

*Median MCQ scores improved from 5.0 to 7.0 out of 8*.

**TABLE 3:** Pre‐ and post‐session MCQ scores (*n* = 32 matched pairs).

Measure	Pre‐session	Post‐session	*p*
Median score out of 8 (IQR)	5.0 (4.0–6.0)	7.0 (6.0–7.2)	< 0.001
Mean score out of 8 (SD)	5.00 (1.48)	6.69 (1.03)	< 0.001
Median improvement (IQR)	—	2.0 (1.0–2.0)	—
Effect size (Cohen's *d*_*z*_)	—	1.12	—

*Note:* Pre‐ and post‐session scores compared using Wilcoxon signed‐rank test (score differences not normally distributed, Shapiro–Wilk *p* = 0.044). IQR, interquartile range; SD, standard deviation; Cohen's *d*_*z*_, effect size for paired comparisons calculated as mean difference divided by the standard deviation of differences. — = not applicable.

## 4 Implications

This programme provides preliminary support for near‐peer, case‐based anatomy teaching as a promising approach for improving MCQ performance and confidence during the pre‐clinical to clinical transition. Cognitive and social congruence may minimise the perceived gap between students and facilitators, enabling questioning and explanations closely aligned with students' existing knowledge. This may mitigate the ‘expert blind spot’, where senior faculty's extensive specialised knowledge can overlook the needs of juniors [10]. Near‐peer facilitators may be well‐placed to model clinical reasoning at a level accessible to transitioning students. Unlike faculty‐led teaching, which can increase perceived hierarchy and power differentials [11], NPT may reduce these barriers while maintaining clinical relevance. In a case‐based setting, where students are expected to actively participate by sharing opinions and asking questions [12], the psychologically safe environment described by students likely reinforced willingness to engage and participate.

Together, these findings suggest that combining near‐peer facilitation with case‐based teaching may have particular value during the transition to clinical practice. They also provide a plausible illustration of how cognitive and social congruence and psychological safety may interact within near‐peer, case‐based teaching to support the application of pre‐clinical anatomical knowledge to clinical scenarios. However, the extent to which these mechanisms operated independently or interactively remains difficult to separate from this programme design.

*Combining near‐peer facilitation with case‐based teaching may have particular value during the transition to clinical practice*.

Facilitators also reported benefits consistent with existing literature on the reciprocal value of NPT [13], particularly the confidence gained in the material prior to their own exams. The requirement to understand material sufficiently to teach it may itself represent an additional learning benefit by encouraging deeper engagement. This reciprocal value may represent a key strength of this programme, helping to address concerns about reduced anatomy exposure for both junior and senior clinical students. However, a formal qualitative evaluation was outside the scope of this programme, and therefore, these findings should be interpreted cautiously, being presented as illustrative rather than formal qualitative findings. Findings may also be subject to response bias given facilitators' involvement in programme design. Further exploration using formal qualitative methods would be warranted in future iterations.

The single‐institution, opt‐in nature of this programme limits generalisability. Participation was voluntary, and sessions were fully subscribed, raising the possibility of self‐selection bias; students with greater interest in anatomy or extracurricular learning may have been more likely to attend, potentially leading to an overestimate of effectiveness in a general student population. The absence of a comparison group means alternative explanations for observed improvements, including content familiarity or testing effects, cannot be excluded. Of these, testing effects are perhaps the most plausible given students' exposure to pre‐session MCQs immediately before teaching [14].

Parallel rather than identical MCQ sets were used to mitigate this, though score improvements may partly reflect differences in question difficulty. Faculty familiar with undergraduate assessment design reviewed both sets for consistency, but formal equivalence testing was not undertaken. Future iterations should address this to more robustly attribute score improvements to knowledge acquisition rather than assessment variability. Outcomes were measured immediately post‐session, capturing short‐term acquisition rather than sustained learning. Research consistently demonstrates that immediate gains do not reliably predict long‐term retention [15], and future iterations should incorporate delayed follow‐up assessments to better evaluate long‐term gains.

Near‐peer, case‐based anatomy teaching may represent a valuable approach to supporting the pre‐clinical to clinical transition within undergraduate medical education. Future research should expand implementation across institutions, assess longer‐term retention and further evaluate the mechanisms underpinning effectiveness.

## Author Contributions

**O. Rackham:** conceptualization, investigation, methodology, data curation, formal analysis, project administration, visualization, resources, writing – original draft, writing – review and editing. **M. M. Maitland:** conceptualization, methodology, data curation, investigation, formal analysis, project administration, resources, writing – review and editing. **P. Ahluwalia:** conceptualization, methodology, data curation, investigation, formal analysis, project administration, resources, writing – review and editing. **O. Murray:** methodology, validation, supervision, resources, writing – review and editing. **J. E. McKay:** writing – review and editing, resources, supervision, methodology, validation.

## Funding

The authors have nothing to report.

## Ethics Statement

This project received ethical approval from the Medical Education Research Ethics Committee (MEREC), University of Edinburgh (Project reference number: 2026‐29).

## Consent

Formal written consent was not obtained; however, implied consent was considered sufficient given the nature of the study. Participants demonstrated willingness to engage by voluntarily completing the MCQ assessments and feedback questionnaires. No personally identifiable data were collected or reported.

## Conflicts of Interest

The authors declare no conflicts of interest.

## Data Availability

The anonymised data supporting the findings of this study are available from the corresponding author upon reasonable request. There are no other current studies using this data set.
